# Removal possibilities of colloidal chromium (III) oxide from water using polyacrylic acid

**DOI:** 10.1007/s11356-012-1273-6

**Published:** 2012-11-06

**Authors:** Małgorzata Wiśniewska, Katarzyna Szewczuk-Karpisz

**Affiliations:** Department of Radiochemistry and Colloids Chemistry, Faculty of Chemistry, Maria Curie Sklodowska University, M. Curie Sklodowska Sq. 3, 20-031 Lublin, Poland

**Keywords:** Water purification, Colloidal impurities, Polymeric flocculation, Solid particles destabilization, Chromium (III) oxide stability, Polyacrylic acid adsorption

## Abstract

The lack of water is the most serious threat to humanity that leads to more efficient water and sewage treatment. Currently, many scientists are looking for new coagulants, flocculants and physicochemical methods allowing for sufficient removal of pollutants from water. The presence of various types of pigments, including chromium (III) oxide, poses the major problem. Even small amounts of these substances inhibit life processes in water. In this paper, the stability of Cr_2_O_3_ suspension in the absence and the presence of polyacrylic acid (PAA) was determined. To explain the changes in the system stability, the adsorption and electrokinetic measurements were performed. The chromium (III) oxide suspension not containing PAA is the most stable at pH = 3. Under these conditions, each positively charged solid particle is surrounded by a negatively charged diffusion layer which protects from particle collision and aggregates formation (electrostatic stabilization). In turn, the Cr_2_O_3_ suspension containing the PAA is most unstable also at pH = 3. In this case, the polymer causes destabilization of the colloidal suspension, which results from charge neutralization of solid particles by adsorbed PAA.

## Introduction

Nowadays, the lack of water is a significant problem in many countries. In developing countries, e.g. India, this is mainly caused by the rain outflow, which is too quick for efficient use. In 1995, 436 million people in 29 countries experienced water deficit. However, the World Bank estimates that by 2025, the number may rise to 1.4 billion in 48 countries (Parliamentary Office of Science and Technology 2002). Poland has the poorest drinking water resources among all European countries because of its climatic and hydrologic conditions. Relatively low rainfall and direct flow of groundwater to the Baltic Sea are responsible for the formation of water deficit areas in many Polish regions (Skośkiewicz 1996). Moreover, the phenomenon of discharge of untreated or just mechanically treated sewages to rivers decreases the amount of available water, which could have been used in the food industry and transformed the Baltic Sea from oligotrophic to strong eutrophic type over the last century (Main Inspectorate of Environmental Protection 2011). Therefore, effective water and sewage treatments have become increasingly important in recent years.

Physicochemical composition of surface water contrary to that of groundwater is highly dependent on human activities. Anthropogenic pollution increases diversity and variability of the percentage of each chemical compound in the surface water and destroys many natural ecosystems. What is more, water pollutants have usually negative impact on the human body (Kowal and Świderska-Bróż 2009). Therefore, the interest in water contamination is continually growing, the number of standardized indicators of water quality is getting more numerous and there is a high tendency to reduce the permissible levels of micropollutants to those at which they do not cause pathological changes in consumers (Świderska-Bróż 1993). The amount of surface water impurities varies in wide ranges, i.e. from traces to several grammes per cubic metre. There are primary pollutants, which occur in natural water and secondary ones, formed during the water treatment (Dojlido 1987). The uncontaminated water is dominated by inorganic cations and anions derived from rocks and soils forming the catchment. The increasing content of organic substances is the evidence for gradual water pollution. Another indicator of undesirable chemicals in water may be aquatic organisms whose presence depends on water quality (Kaleta and Puszkarewicz 2011).

Most of the water pollutants are colloids characterized by the particle size from 1 to 1,000 nm and the large surface area from 100 to 5,000 m^2^/g (Sontag 1982). They usually have a negative electric charge, which comes from hydroxide ions. In the water solution, a double electrical layer is formed around each particle. Reduction of the difference between the adsorption layer potential and that of the moving part of diffusion layer, i.e. the zeta potential (ξ) to the value close or equal to zero leads to the weakening of repulsive forces between particles. Under such conditions, complete coagulation of particles, i.e. destabilization of colloidal system, is possible. Coagulation reduces the system dispersion and helps flocculation process, which is mainly based on the formation of colloidal particle aggregates in the presence of a polymer (Basiński 1965; Adamski 2002).

The presence of inorganic ions in water is often caused by ineffective purification of industrial wastewaters. These chemicals are especially dangerous due to both their toxic and possibly carcinogenic nature, persistence and difficult biodegradation (Ali 2010). Inorganic ions speciation poses a serious problem of their disposal and removal. Fortunately, the adsorption process may give the opportunity to remove them from water solution (Ali and Gupta 2006). This way of water and wastewater treatment can be carried out using active carbon or its inexpensive substitutes, e.g. industrial wastes such as bagasse fly ash—a sugar industry waste which allows to lead and chromium removal (Ali 2012; Gupta and Ali 2004) and can be used as adsorbent during zinc removal from water solution (Gupta and Sharma 2003). Acquisition of adsorbents from wastes can significantly reduce the cost of water treatment while the high efficiency is preserved. Activated carbon developed from waste rubber tires (Gupta et al. 2012b) and other low-cost fertilizer industry waste material (Gupta et al. 2010) allow chromium removal. Moreover, thanks to waste carbon flurry, defluoridation is possible (Gupta et al. 2007a, b, c).

In recent years, scientists from around the world worked on the synthesis of adsorbents, which allow removal of certain inorganic ions from water. In the literature, the adsorbents applied to water treatment were often synthesized by a combination of activated carbon and mineral oxides. The magnesium dioxide-coated multiwall carbon nanotube nanocomposite can be used to lead (II) removal (Saleh and Gupta 2012a), and the manganese oxide and multiwall carbon nanotube combination (MWCNT/MnO2) allows pre-oxidation of As (III) to As (V) and arsenate sorption simultaneously (Saleh et al. 2011). What is more, the adsorbent made of iron oxide and carbon nanotubes can be used to chromium removal (Gupta et al. 2011a) and the alumina-coated carbon nanotubes can be extremely helpful in lead removal (Gupta et al. 2011b). The use of composite adsorbent, i.e. produced by combining mineral oxide and activated carbon, allows for more efficient removal of impurities from water and wastewater. The appropriate flow rate of feed solution makes the contact time larger and, hence, the amount of adsorbed ions is higher. Sometimes the biosorbents, such as *Oedogonium hatei* (Gupta and Rastogi 2009) and *Ficus carica* (Gupta et al. 2012c) are used for inorganic ions separation.

Adsorption may be also used to removal of organic substances, including dyes, which are highly undesirable in water (Jain et al. 2003; Gupta et al. 2007b). The composite catalyst consisting of multiwalled carbon nanotubes and titanium dioxide can be used to photo-catalyzed degradation of dye methyl orange (Saleh and Gupta 2012b) and the titanium dioxide catalyst allows to photochemical degradation of safranin-T (Gupta et al. 2007a). Using the adsorption process also enables the removal of erythrosine (Gupta et al. 2006) and chlorophenols (Jain et al. 2004). The universal nature, ease of operation and high efficiency of the adsorption process could make adsorption really advantageous and widely applied in water treatment (Ali et al. 2012; Gupta et al. 2012a).

Sedimentation, coagulation, filtration and flocculation are frequently used in water and wastewater treatment to colloid particle removal. These processes allow separation of all colloidal micropollutants that give water undesirable colour and turbidity (Masschelein 1985; Montgomery 1985). Chromium (III) oxide is a fine crystalline, amphoteric, green solid which is the most common form of chromium in the environment (Barnhart 1997). The prevalence of chromium (III) oxide is caused by the tendency of particular forms of chromium to turn into oxide due to the interaction of the various elements of the environment. Cr_2_O_3_ is not thermodynamically stable in the environment; however, its reactivity is low. This mineral oxide takes ninth place among the most prevalent compounds in the earth’s crust. However, the green color is rarely seen due to the masking by other compounds present in minerals. Chromium (III) oxide is the most stable, non-toxic green dye, widely used in many industries such as plastic, construction, glass and ceramic industries. Therefore, Cr_2_O_3_ is often present in the effluents from these industries, which is highly undesirable (Gettens and Stout 1966). Even small amounts of colourful substances in water impede the penetration of light into its deeper layers, thus inhibiting photosynthesis and other life processes (Filipkowska et al. 2011). Therefore, in this paper, the mechanism of stabilization/destabilization of chromium (III) oxide suspension in the absence and the presence of polyacrylic acid has been determined. It could be helpful in the development of Cr_2_O_3_ removal during water treatment. In the literature, methods of chromium (III) oxide removal from water are very rarely described, which also led the authors to develop the above problem. Polyacrylic acid was chosen because of its ability to flocculate various mineral oxides (e.g. alumina), which was identified during previous studies carried out in the Department of Radiochemistry and Colloids Chemistry UMCS. The authors attempted to examine polyacrylic acid (PAA) behavior to chromium (III) oxide suspension. To understand the mechanism of stabilization/destabilization of Cr_2_O_3_ suspension with and without PAA, the stability, adsorption and electrokinetic measurements were performed.

## Experiment

### Materials

Chromium (III) oxide, Cr_2_O_3_, produced by POCh, was used as the adsorbent in the experiments. Due to the contamination by inorganic ions, the oxide was washed by doubly distilled water to achieve the supernatant conductivity less than 2 μS/cm. After drying, chromium (III) oxide was crushed in a porcelain crucible. The adsorbent specific surface area, determined by the BET method (analysis of nitrogen adsorption–desorption isotherms), was 7.12 m^2^/g.

PAA, the anionic polymer, produced by Aldrich, with the following weight average molecular weights ($\overline{M_w}$): 2,000, 240,000, was used in the study. This polymer contains one type of functional groups in its macromolecule, i.e. carboxylic groups. Those groups may dissociate depending on the pH of the solution according to the following molecular equation:

1 $$ {{\left[ {\begin{array}{*{20}c} {-\mathrm{C}{{\mathrm{H}}_2}} & \text{---} & {\mathrm{CH}-} \\ {} & {} & {_{|}} \\ {} & {} & {\mathrm{COOH}} \\ \end{array}} \right]}_{\mathrm{n}}}\overset{{{{\mathrm{H}}_2}\mathrm{O}}}{\leftrightarrows}{{\left[ {\begin{array}{*{20}c} {-\mathrm{C}{{\mathrm{H}}_2}} & \text{---} & {\mathrm{CH}-} \\ {} & {} & {_{|}} \\ {} & {} & {\mathrm{CO}{{\mathrm{O}}^{-}}} \\ \end{array}} \right]}_{\mathrm{n}}}+{{\mathrm{H}}^{+}} $$

where the pK_a_ value for the polyacrylic acid is equal to 4.5 (Gebhardt and Furstenau 1983). It means that half of the carboxylic groups in the PAA macromolecules is dissociated at pH = 4.5. The following equations:

2 $$ \mathrm{pH}-\mathrm{p}{{\mathrm{K}}_{\mathrm{a}}}=\frac{{\left[ {\mathrm{RCO}{{\mathrm{O}}^{-}}} \right]}}{{\left[ {\mathrm{RCOOH}} \right]}} $$

3 $$ \mathrm{pH}-\mathrm{p}{{\mathrm{K}}_{\mathrm{a}}}=\log \frac{\propto }{{1-\propto }} $$

allowed to calculate the concentration ratio of the –COOH groups to –COO^−^ groups and the degree of dissociation (*α*_d_) at different pH values. At pH <4.5, the number of undissociated groups (−COOH) is greater than that of dissociated ones (−COO^−^), whereas at pH >4.5 the ratio is reversed. At pH = 3, the degree of carboxylic group dissociation is equal to 0.03, at pH = 6, *α*_d_ = 0.96. Almost all carboxylic groups in the PAA macromolecules are dissociated at pH = 9 (*α*_d_ = 0.99) (Wiśniewska and Chibowski 2005).

### Methods

All measurements were performed at 25 °C. Sodium chloride (1 × 10^−2^ mole/dm^3^) was used as the supporting electrolyte.

#### Stability measurements

Stability measurements were made using a turbidimeter Turbiscan Lab^Expert^ which was connected to the cooling module TLab Cooler and specialized computer software. The measurement of single sample lasted 15 h, during which relevant data were recorded every 15 min. The studies were carried out at three pH values, i.e. 3, 6 and 9.

The suspensions without PAA were prepared by adding 0.02 g of chromium (III) oxide to 20 cm^3^ of supporting electrolyte. The samples with PAA were prepared by adding 0.02 g of Cr_2_O_3_ to 20 cm^3^ of solution containing appropriate volume of PAA that gave the final concentration of 100 or 500 ppm and 2 cm^3^ of the supporting electrolyte. Every suspension was sonicated for 3 min. After adjusting the required pH value of the samples, the changes of their stability were examined.

The obtained results are presented as the curves of transmission and backscatter of light passing through the sample. Moreover, the specialized computer software connected to a turbidimeter calculated the Turbiscan Stability Index (TSI) that is very useful in the system stability estimation. TSI is a statistical quantity which takes into account all the processes occurring in the sample, e.g. thickness of the sediment and clean layer, rate of solid particles sedimentation. A computer software calculated the TSI value using the following equation:

4 $$ \mathrm{TSI}=\sqrt{{\frac{{\sum\nolimits_{i=1}^n {{{{\left( {x_i -{x_{\mathrm{BS}}}} \right)}}^2}} }}{n-1 }}} $$

where *x*_*i*_ is the average backscatter for every minute of measurement, *x*_BS_ is the average *x*_*i*_ value, and *n* is number of scans.

TSI is in the range from 0 to 100. The higher the TSI value, the lower the stability of the suspension.

Using a computer software working with a turbidimeter, the average size of Cr_2_O_3_ aggregates or its flocs formed in the presence of the polymer, and their average migration ratio was obtained. What is more, the software calculated these values during the biggest changes in the suspension stability.

#### Adsorption measurements

Adsorption measurements were performed by the static method in the PAA concentration range of 5–500 ppm, at three pH values, i.e. 3, 6 and 9. The concentration of PAA in the samples was determined using the Crummet and Hummel method based on the reaction of polymer with hyamine 1622 (Crummet and Hummel 1963). The obtained turbidity was measured with the UV–Vis Specord M42 spectrophotometer (Carl Zeiss Jena) at 500 nm.

First, the calibration curve, i.e. the dependence of absorbance on the PAA concentration in the sample (ABS = *f*(*C*_PAA_)), was obtained. Then 0.2 g of chromium (III) oxide was added into Erlenmeyer flasks containing 10 cm^3^ of polymer solutions with fixed concentrations. The solid weight was determined taking its surface area into account. After adjusting the pH value in the samples, they were shaken in water bath for 24 h. Then, the sediments were centrifuged and 5 cm^3^ of the supernatant was taken for analysis. The amount of adsorbed polymer was obtained from the difference between the polymer concentration before and after the adsorption process. The single result was the average of five measurements. The measurement error did not exceed 5 %.

#### Electrokinetic measurements

Using potentiometric titrations, the surface charge density of chromium (III) oxide was determined. These measurements were made in the polymer concentration range of 0.01–100 ppm. Of the Cr_2_O_3_, 1.5 g was added to a thermostated teflon vessel containing 50 cm^3^ of supporting electrolyte solution or PAA solution with a fixed concentration. Then 0.2 cm^3^ of HCl solution with a concentration of 0.1 mole/dm^3^ was added to the vessel, which gave the solution an initial pH in the range of 3–3.5. The suspensions were titrated with NaOH solution with a concentration of 0.1 mole/dm^3^. Potentiometric titrations were made using the following apparatuses: thermostat RE 204 (Lauda), glass and calomel electrode (Beckman Instruments), pH meter PHM 240 (Radiometer), automatic microburette Dosimat 765 (Metrohm) connected to the computer and printer. The surface charge density of Cr_2_O_3_ was obtained from the specialized program Titr_v3 which was elaborated by W. Janusz.

The zeta potential measurements were performed in the PAA concentration range of 0.01–100 ppm and pH value range of 3–10 ± 0.1–0.03 g of the solid was added to the supporting electrolyte solution or PAA solution with a fixed concentration. After the suspension sonification (3 min), the required pH value in the samples was adjusted. Before the measurement, the measuring apparatus was washed twice by the analyzed suspension. The electrokinetic potential was measured with the Zetasizer 3000 zeta meter (Malvern Instruments) connected to the computer. A single result was the average of five repetitions. The measurement error did not exceed 3 %.

## Results and discussion

### Stability of Cr_2_O_3_ suspension in the absence and the presence of PAA

**Fig. 1:**
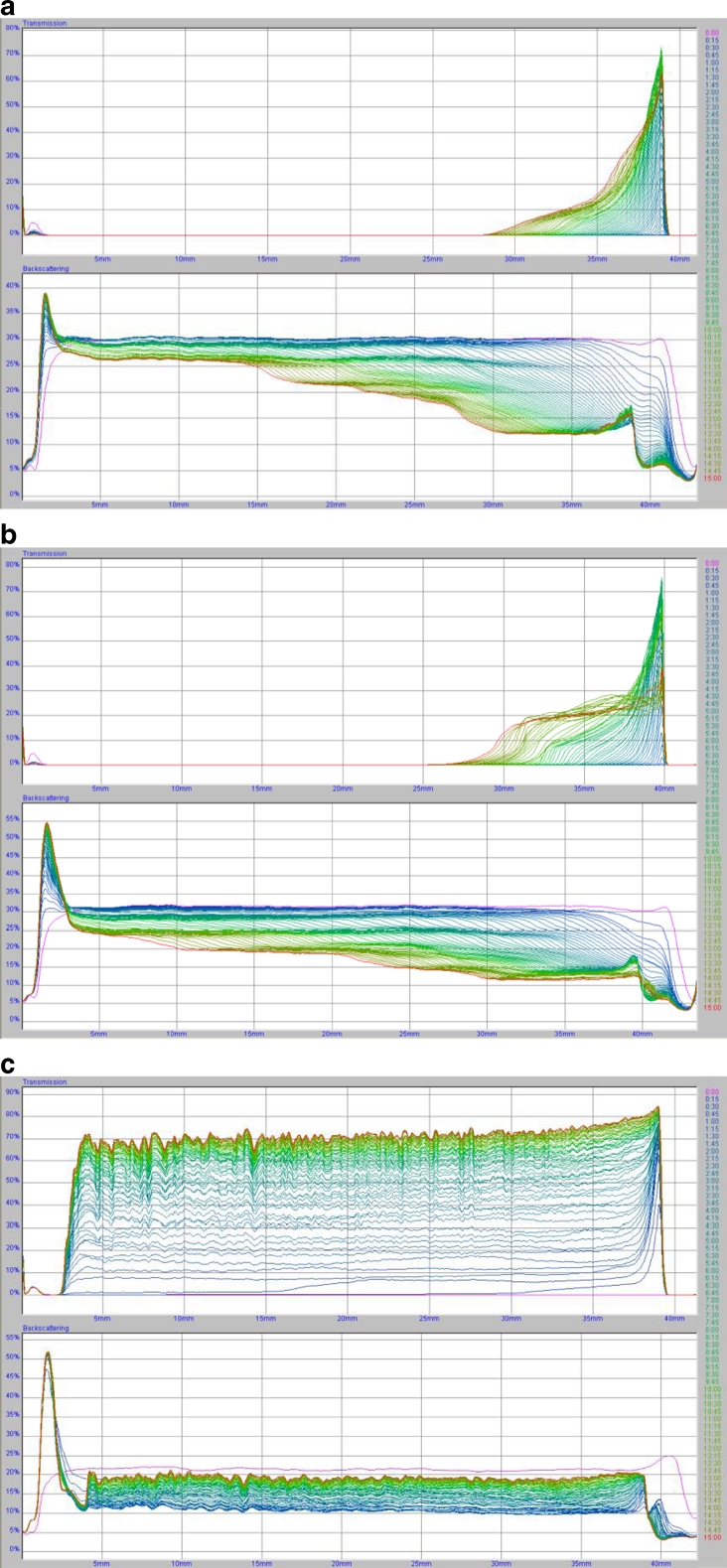
The transmission and backscatter curves for systems. **a** Cr_2_O_3_, **b** Cr_2_O_3_–PAA 2,000, **c** Cr_2_O_3_–PAA 240,000, C_PAA_ = 100 ppm, pH = 3. Samples **a** and **b** are characterized by high stability, and sample c is unstable

**Fig. 2:**
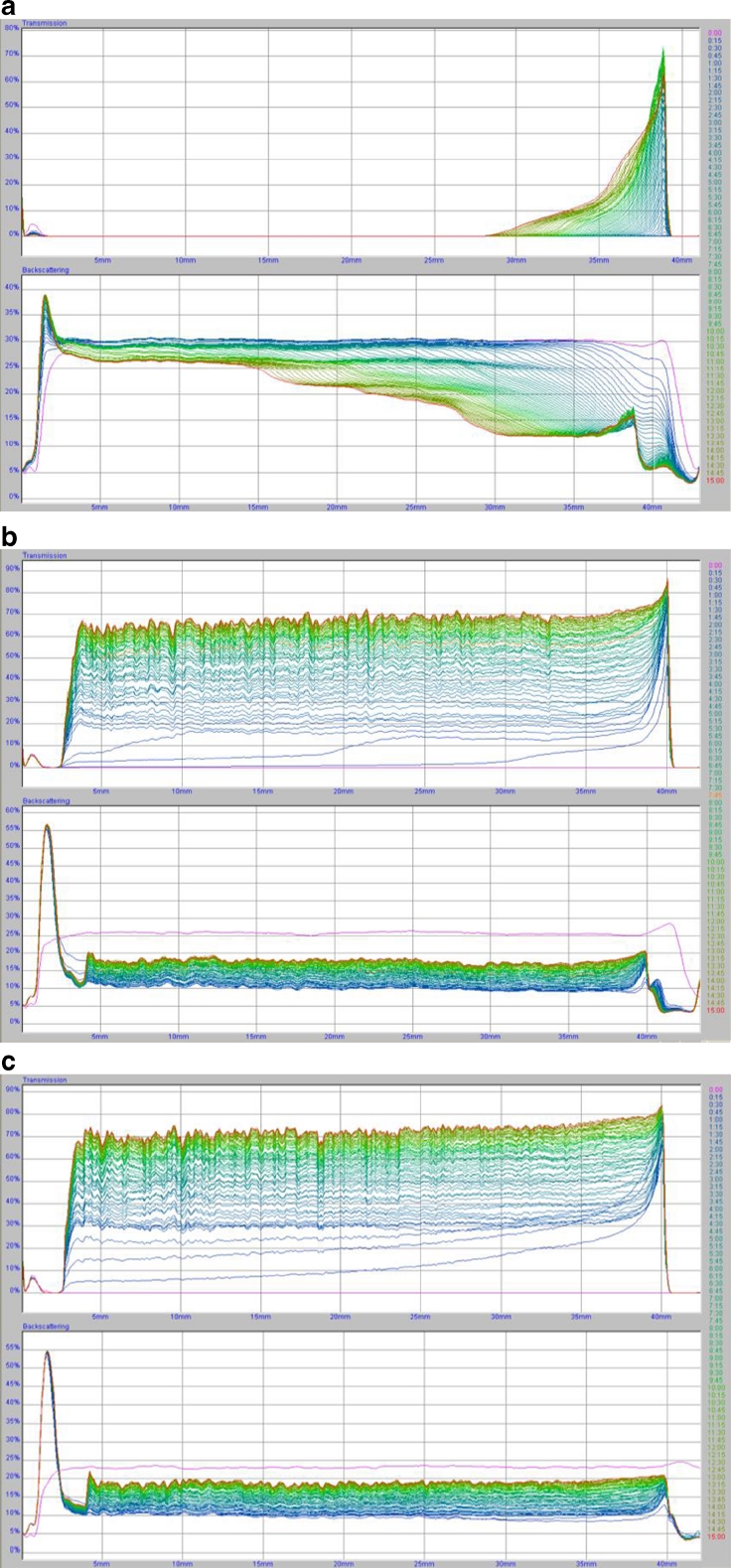
The transmission and backscatter curves for systems. **a** Cr_2_O_3_, **b** Cr_2_O_3_–PAA 2,000, **c** Cr_2_O_3_–PAA 240,000, C_PAA_ = 500 ppm, pH = 3. Sample **a** is characterized by high stability, samples **b** and **c** are unstable

Stability measurements of chromium (III) oxide suspension in the absence and the presence of polyacrylic acid is needed to determine the optimal conditions for removal of chromium (III) oxide from water. The results were obtained as the curves of transmission and backscatter of light passing through the sample. The low level of transmission and high level of backscatter confirmed the high stability of the system. The lower the system stability is, the higher the transmission and lower backscatter. The clarification process occurring in the analyzed systems is reflected in the peaks on the transmission curves. In turn, the thickness of sediment formed during the measurement corresponds to the peaks on the backscatter curves. The analysis of the arrangement of the curves relative to one another, especially the distances between them, allows the assessment of the dynamics of the processes occurring in the sample during the measurement. A large distance between the curves indicates a lower stability of the suspensions, thus high-speed particle migration and rapid sedimentation. The overlap of the curves indicates a greater stability in the system and a much lower rate of particle sedimentation. The transmission and backscatter curves obtained at 25 °C for the following systems: Cr_2_O_3_, Cr_2_O_3_–PAA 2,000; Cr_2_O_3_–PAA 240,000 at pH = 3 and at two polymer concentrations (100 and 500 ppm) are shown in Figs. 1 and 2.

In addition, the specialized computer software connected to a turbidimeter calculated the TSI value (Table 1), the average size of solid aggregates and its flocs formed in the presence of PAA (Table 2), their average migration ratio (Table 3) for all analyzed systems. Moreover, using the same program, the above values were obtained during the greatest changes in the suspension stability (Table 4).

**Table 1:** The TSI values for chromium (III) oxide suspensions in the absence and the presence of polyacrylic acid

System	Polymer concentration [ppm]	TSI		
		pH = 3	pH = 6	pH = 9
Cr_2_O_3_	–	12.76	61.00	29.28
Cr_2_O_3_–PAA 2,000	100	13.57	11.61	12.30
	500	67.66	12.24	11.26
Cr_2_O_3_–PAA 240,000	100	65.55	12.27	10.63
	500	70.41	9.19	10.15

**Table 2:** The average size of aggregates (flocs) in the suspension of chromium (III) oxide in the absence and the presence of polyacrylic acid at 15 h

System	PAA concentration [ppm]	*d* [μm]		
		pH = 3	pH = 6	pH = 9
Cr_2_O_3_	–	0.077	0.540	0.127
Cr_2_O_3_–PAA 2 000	100	0.083	0.063	0.071
	500	0.553	0.068	0.072
Cr_2_O_3_–PAA 240 000	100	0.156	0.067	0.109
	500	0.569	0.061	0.080

**Table 3:** The average sedimentation rate of aggregates (flocs) in the suspension of chromium (III) oxide in the absence and the presence of polyacrylic acid at 15 h

System	PAA concentration [ppm]	*V* [μm/min]		
		pH = 3	pH = 6	pH = 9
Cr_2_O_3_	–	0.83	40.12	2.23
Cr_2_O_3_–PAA 2,000	100	0.73	0.54	0.68
	500	42.00	0.64	0.71
Cr_2_O_3_–PAA 240,000	100	3.33	0.63	1.64
	500	44.56	0.52	0.87

**Table 4:** The list of parameters characterizing the suspension of chromium (III) oxide in the absence and the presence of polyacrylic acid during the biggest changes in the system stability

System	PAA concentration [ppm]	*t* [h]		
		*d* [μm]		
		*V* [μm/min]		
		pH = 3	pH = 6	pH = 9
Cr_2_O_3_	–	t = 1	t = 4	t = 2
		*d* = 0.221	*d* = 0.98	*d* = 0.154
		*V* = 6.74	*V* = 132.3	*V* = 3.27
Cr_2_O_3_–PAA 2,000	100	*t* = 1	*t* = 1	*t* = 1
		*d* = 0.318	*d* = 0.178	*d* = 194
		*V* = 7.88	*V* = 4.33	*V* = 5.18
	500	*t* = 5	*t* = 1	*t* = 1
		*d* = 0.941	*d* = 0.186	*d* = 0.17
		*V* = 121.65	*V* = 4.75	*V* = 3.97
Cr_2_O_3_–PAA 240,000	100	*t* = 1	*T* = 1	*t* = 1
		*d* = 0.46	*d* = 0.195	*d* = 0.211
		*V* = 29.25	*V* = 5.20	*V* = 6.10
	500	t = 4	t = 1	t = 1
		d = 1.1	d = 0.153	d = 0.204
		*V* = 165.1	*V* = 3.23	*V* = 5.71

The analysis of TSI values (Table 1) showed that the Cr_2_O_3_ suspension not containing PAA has the lowest stability at pH = 6 (TSI = 61.00), whereas at pH = 3 this system is characterized by relatively high stability (TSI = 12.76). At pH = 9, the average TSI value was obtained (TSI = 29.28). Less stability of the system is equivalent to the formation of large Cr_2_O_3_ aggregates and high speed of their sedimentation. In stable suspensions, smaller aggregates of solid particles are formed, and their migration ratio is significantly slower (Tables 2 and 3). Of the Cr_2_O_3_ suspensions containing PAA 2,000, the system with the polymer concentration of 500 ppm at pH = 3 has the lowest stability. In turn, of the Cr_2_O_3_ suspensions containing PAA 240,000, the systems with the PAA concentrations of 100 and 500 ppm at pH = 3 are the least stable. It was also found that in low stable samples, the formed flocs are larger, and their migration rate is higher than in highly stable samples. For instance, in the Cr_2_O_3_ system with PAA 2,000 of the concentration of 500 ppm at pH = 3, the average size of flocs was equal to 0.553 μm and they fall down with the average speed of 42 μm/min while in the same system at pH = 6 the average size of flocs was 0.068 μm and the average rate of sedimentation was 0.64 μm/min.

In most analyzed systems, the greatest changes in stability occurred after 1 h (Table 4). The exceptions were the systems at pH = 3 containing the polymer of the concentration of 500 ppm, where the biggest changes in stability occurred after 4–5 h. Besides, in the systems without the polymer at pH = 6 and pH = 9, the maximum of changes in stability was observed after 1 and 2 h, respectively.

### Adsorption properties of poly(acrylic acid) on the chromium (III) oxide surface

**Fig. 3:**
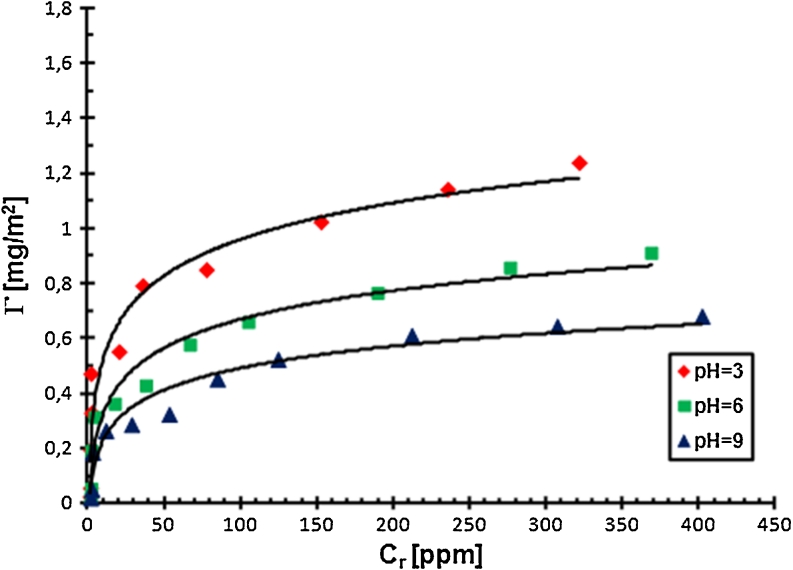
Adsorption isotherms of PAA 2,000 on the Cr_2_O_3_ surface at different pH values

**Fig. 4:**
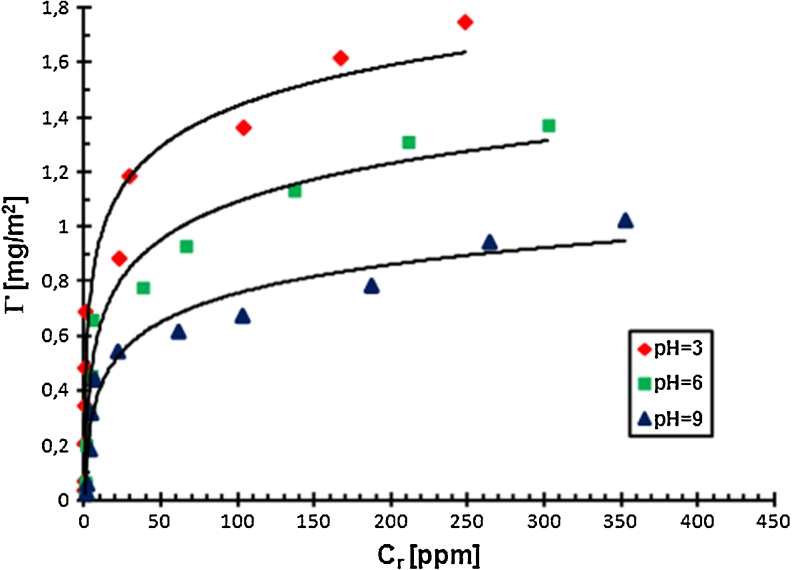
Adsorption isotherms of PAA 240,000 on the Cr_2_O_3_ surface at different pH values

To explain the determined changes in the stability of Cr_2_O_3_ suspension in the absence and the presence of PAA and to obtain the preliminary information about the conformation of PAA macromolecules at the solid–liquid interface, the adsorption measurements were made. The results are shown as the adsorption isotherms in Figs. 3 and 4.

The analysis of the adsorption isotherms indicated that the amount of PAA adsorbed on the Cr_2_O_3_ surface is growing with the increasing PAA concentration. In this case, the adsorption growth is mainly related to the adsorption of macromolecules of larger molecular weight. Using polyacrylic acid has a low degree of polydispersity. The ratio of the weight average molecular weight and the number average molecular weight, i.e. polydispersity coefficient (*D*), for PAA is approximately 1.4. It means that the molecular weights of PAA macromolecules in the sample are not identical, they change in a specific range. At low PAA concentrations, practically all polymer macromolecules, i.e. having smaller and larger molecular weights, absorb on the solid surface, whereas at higher PAA concentrations, shorter PAA chains are displaced from the metal oxide surface by longer ones. This is related to the greater affinity of longer chains for the solid surface, probably due to higher absolute value of free adsorption energy of the polymer with higher molecular weight.

**Fig. 5:**
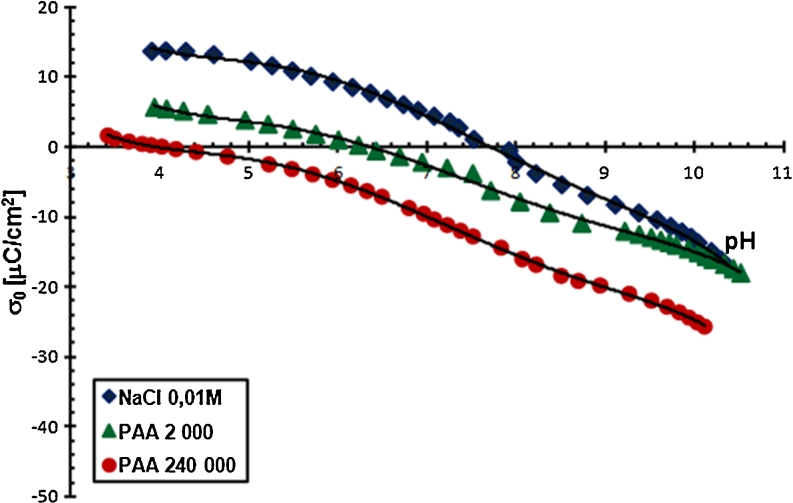
Density of the Cr_2_O_3_ surface charge with and without PAA, C_PAA_ = 0.01 ppm

**Fig. 6:**
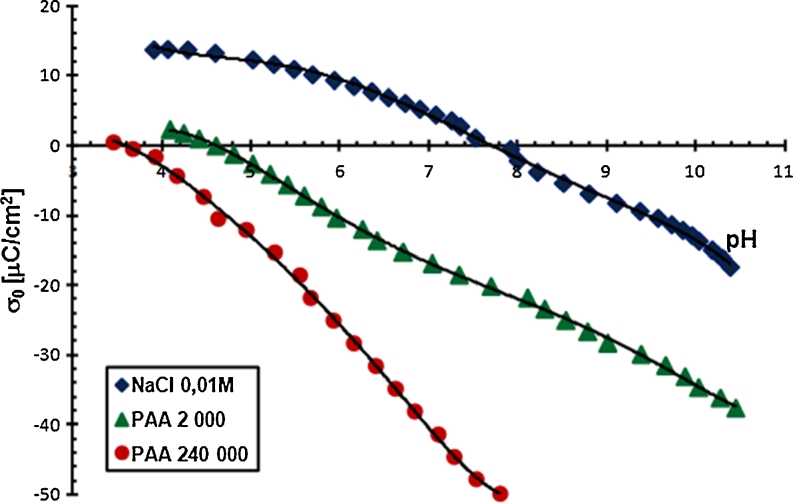
Density of the Cr_2_O_3_ surface charge with and without PAA, C_PAA_ = 100 ppm

pH is a parameter significantly affecting the amount of the PAA adsorption on the Cr_2_O_3_ surface. The obtained results showed that the amount of polymer adsorbed on the solid surface decreases with the increasing pH value. It is related to the increasing carboxylic groups ionization in the PAA macromolecules and the change in density and charge on the adsorbent surface. As mentioned previously, in the PAA chains, at pH <4.5 undissociated carboxylic groups dominate. In turn, at pH >4.5, dissociated carboxylic groups are prevalent. Moreover, potentiometric titrations, which will be described in detail in the next section, showed that pH_pzc_ for chromium(III) oxide is 7.6 (Figs. 5 and 6).

Thus, at pH = 3 there is electrostatic attraction between the polymer and the positively charged Cr_2_O_3_ surface in the system. In addition, due to the small number of COO^−^ groups in the PAA macromolecules (*α*_d_ = 0.03), the chains absorbed on the solid surface have a more coiled conformation. This PAA structure gives the possibility of the adsorption of a larger number of macromolecules on the unit adsorbent surface, which results in the highest polymer adsorption under these conditions.

At pH = 6, a significantly greater number of dissociated carboxylic groups in PAA macromolecules (*α*_d_ = 0.96) causes the repulsion between individual polymer segments, so that the PAA chains have more expanded conformation. Adsorption of these macromolecules blocks the active sites on the Cr_2_O_3_ surface and makes them unavailable to the other PAA chains. As a result, the amount of the polymer adsorbed at pH = 6 is smaller than at pH = 3. Furthermore, the density of positive surface charge of solid particles at pH = 6 is smaller than at pH = 3, which affects the decrease of PAA adsorption too.

At pH = 9, the degree of PAA chain expansion is even greater than at pH = 6 because of the dissociation of almost all carboxylic groups in polymer macromolecules (*α*_d_ = 0.99). Additionally, under these conditions the chromium (III) oxide surface is negatively charged. The repulsion between the negative surface of metal oxide particles and negatively charged polymer segments should exclude the adsorption of anionic PAA on the Cr_2_O_3_ surface. However, the measurement results showed that at pH = 9, a small amount of polymer adsorbed on the solid surface. Thus, some other interactions causing adsorption must be present in the system. These are hydrogen bridges, which are formed between the PAA carboxylic groups and Cr_2_O_3_ surface groups. All types of Cr_2_O_3_ surface groups, i.e. –CrOH, –CrOH_2_^+^, –CrO^−^, and both types of PAA carboxylic groups, i.e. dissociated and undissociated can participate in the formation of these bridges. This indicates that the hydrogen bonding between the adsorbent and the adsorbate in the system is formed in the whole pH value range, not only at pH = 9. However, the occurrence of PAA adsorption at pH = 9 in the strong electrostatic repulsion conditions is clear evidence for their presence.

The increase in the amount of PAA adsorbed on the solid surface with the increase in PAA weight average molecular weight is related to the different affinity of PAA macromolecules of different lengths to the adsorbent surface. The number of polymer segments interacting with the solid surface can be identical in the case of PAA 2,000 and PAA 240,000. However, on the Cr_2_O_3_ surface, the PAA 240,000 chains have conformation with more loops and tails compared to PAA 2,000. That is why the increase in the amount of adsorbed polymer with the increase in its molecular weight is obvious. Shorter chains have flatter conformation on the solid surface and block active sites on it. This fact leads to smaller adsorption of PAA macromolecules with lower molecular weights.

### Effect of PAA adsorption on the double electrical layer structure on the surface of Cr_2_O_3_ particles

#### Density of Cr_2_O_3_ surface charge in the absence and the presence of PAA

The density of chromium (III) oxide surface charge and pH at which this charge is equal to 0 (pH_pzc_) allows to determine the structure of the double electrical layer at the chromium (III) oxide/supporting electrolyte solution (polymer solution) interface. The Cr_2_O_3_ surface charge formed by the reaction of its hydroxyl groups with liquid phase components was determined by potentiometric titration. The results are shown in Figs. 5 and 6.

Potentiometric titration of Cr_2_O_3_ suspensions without PAA allows for the determination of pH_pzc_, i.e. the pH value at which the total Cr_2_O_3_ surface charge is 0. This value for chromium (III) oxide is equal to about 7.6. Then the concentrations of positive and negative surface groups are identical. At pH below pH_pzc_, the Cr_2_O_3_ surface is positive, whereas at pH above pH_pzc_, negative. The polymer addition shifts the Cr_2_O_3_ pH_pzc_ value towards the acidic pH value. Moreover, PAA adsorption causes the reduction of Cr_2_O_3_ surface charge, which is even greater, the higher the polymer molecular weight and the higher the pH of the solution are. The growth of these two factors causes the increase in the amount of dissociated carboxylic groups (−COO^−^) in the PAA macromolecules, which are located in loop and tail structures of the adsorbed polymer. The –COO^−^ groups are primarily responsible for surface charge reduction. Only a few polymer segments are directly connected with the solid surface through the –COO^−^ groups. They interact with the hydroxyl groups on the chromium (III) oxide surface and are in the train structure.

A decrease in the Cr_2_O_3_ surface charge with the increasing pH of solution becomes more rapid when PAA concentration is growing. This is probably related to the already mentioned displacement of the shorter polymer chains by the longer ones at higher polymer concentrations. Longer chains contain a larger number of –COO^−^ groups which are not bound with the solid surface. They form the adsorption layer with the segments with many loop and tail structures. It is noteworthy that in the PAA 2,000 and 240,000 solution with a concentration of 500 ppm, the Cr_2_O_3_ particle surface is negative in the whole range.

#### The zeta potential of Cr_2_O_3_ particles in the absence and the presence of PAA

**Fig. 7:**
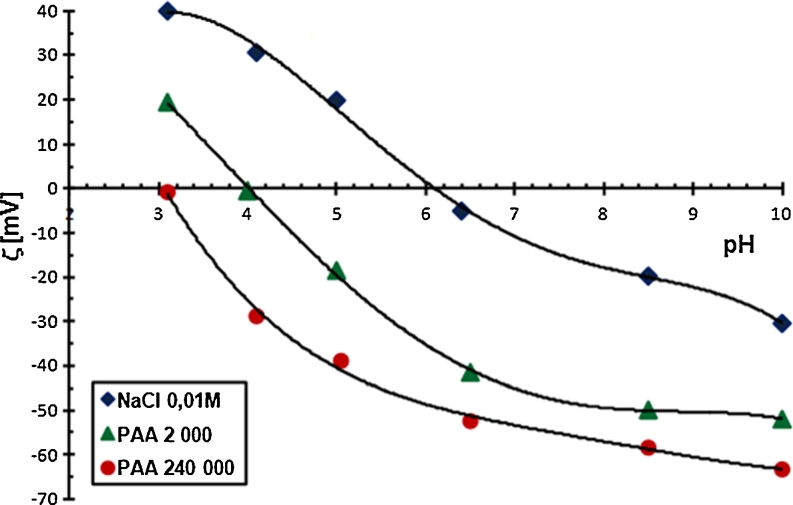
The zeta potential of Cr_2_O_3_ particles with and without PAA, C_PAA_ = 0.01 ppm

**Fig. 8:**
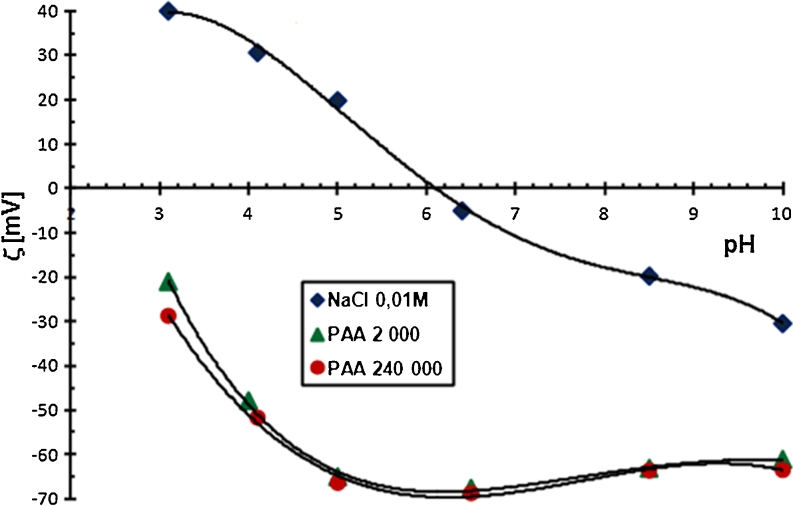
The zeta potential of Cr_2_O_3_ particles with and without PAA, C_PAA_ = 100 ppm

The zeta potential is another parameter characterizing the structure of double electrical layer formed around the solid particles. The results of zeta potential measurements are shown in Figs. 7 and 8.

Based on the analysis of these figures, it was concluded that polyacrylic acid lowers the zeta potential of Cr_2_O_3_ particles. At high concentrations of PAA (Fig. 7), the zeta potential of Cr_2_O_3_ is negative throughout the whole pH range. The electrokinetic potential reduction is mainly due to the dislocation of the slipping plane by the polymer chains in specific conformation. The increase in polymer molecular weight and its concentration is equivalent to the elongation of the loop and tail structures in the adsorbed PAA macromolecules. Vertical arrangement of these structures in relation to the solid particle surface causes the significant shift of slipping plane, which results in significant reduction of zeta potential.

Anionic character of PAA is another factor lowering the zeta potential value. The number of dissociated carboxylic groups in the PAA macromolecules, growing with the increasing pH value, undoubtedly, affects the electrokinetic potential value. These carboxylic groups, present in the diffusion part of double electrical layer in loop and tail structures of the adsorbed chains, lower the zeta potential of Cr_2_O_3_ particles.

A small contribution to the obtained changes in the electokinetic potential of Cr_2_O_3_ particles in the presence of PAA may affect active sites blocking on the Cr_2_O_3_ surface by the adsorbed polymer chains. As a result, active sites become unavailable for the supporting electrolyte ions and other PAA macromolecules. Thus, the arrangement of charges in the diffusion layer and ξ potential value changes. This phenomenon is probable at low concentrations and low molecular weight of PAA because the polymer chains have flatter conformation under these conditions.

To sum up, it can be concluded that the reduction of zeta potential of chromium (III) oxide particle in the presence of PAA is associated with: (1) the presence of dissociated carboxyl groups in the polymer macromolecules, (2) the shift of slipping plane (typical of polymer high concentration and its high molecular weight) and (3) the blockade of the active sites on the solid surface (typical of polymer low concentration and low molecular weight).

The obtained results also showed that pH_iep_, the isoelectric point of the Cr_2_O_3_ surface, in which the number of positive and negative charges on the slipping plane is identical, is equal to 6. The PAA addition to the system moves the pH_iep_ value to a more acidic one. A PAA 2,000 with a concentration of 0.01 ppm reduces pH_iep_ to about 4, while PAA 240,000 with the same concentration reduces this value to about 3.

### Mechanism of stabilization/destabilization of Cr_2_O_3_ suspension in the absence and presence of PAA

Analysis of adsorption and electrokinetic measurement results helped explain the mechanism of stabilization and destabilization of chromium (III) oxide suspension with and without polyacrylic acid.

Cr_2_O_3_ suspensions not containing the polymer are most stable at pH = 3. Under these conditions in the supporting electrolyte solution, the electrostatic stabilization occurs between solid particles. It is provided by the highest value of Cr_2_O_3_ zeta potential, which at pH = 3 is about 40 mV. In this situation, each positively charged particle is surrounded by the diffusion layer of negative charge, which comes from the supporting electrolyte. Thus, the effective barrier protecting from particle collision and Cr_2_O_3_ aggregates formation is made.

At pH = 6, the Cr_2_O_3_ suspension is unstable because the charge of the chromium (III) oxide diffusion layer is nearly 0 under these conditions. This means that the concentrations of positively and negatively charged groups in the slipping plane are identical. The zero charge of the diffusion layer is equivalent to the loss of electrical repulsive forces between particles which allow to form large, rapidly sedimenting aggregates. Thus, at pH = 6, there is a coagulation process of chromium (III) oxide particles in the suspension.

In turn, at pH = 9, Cr_2_O_3_ suspensions have stability intermediate between those obtained at pH = 3 and pH = 6. This is probably related to the intermediate value of Cr_2_O_3_ electrokinetic potential whose absolute value is approximately 20 mV.

**Fig. 9:**
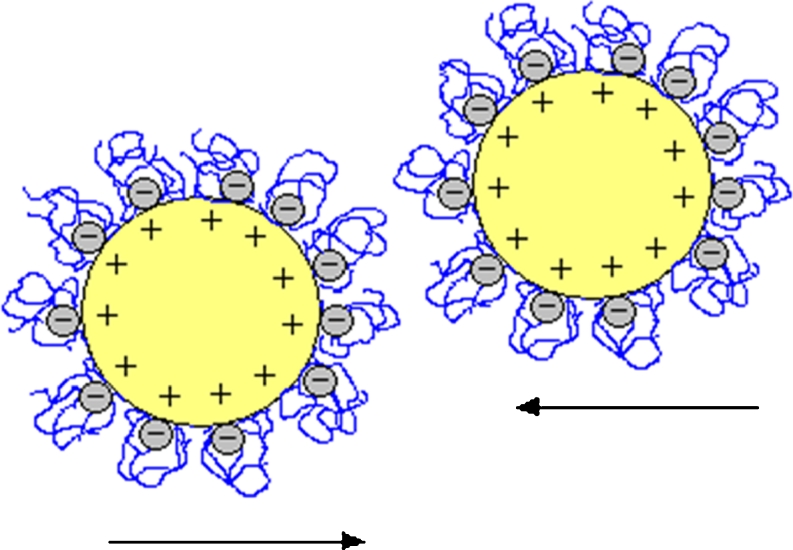
The adsorbent surface charge neutralization by adsorbed PAA chains at pH = 3

**Fig. 10:**
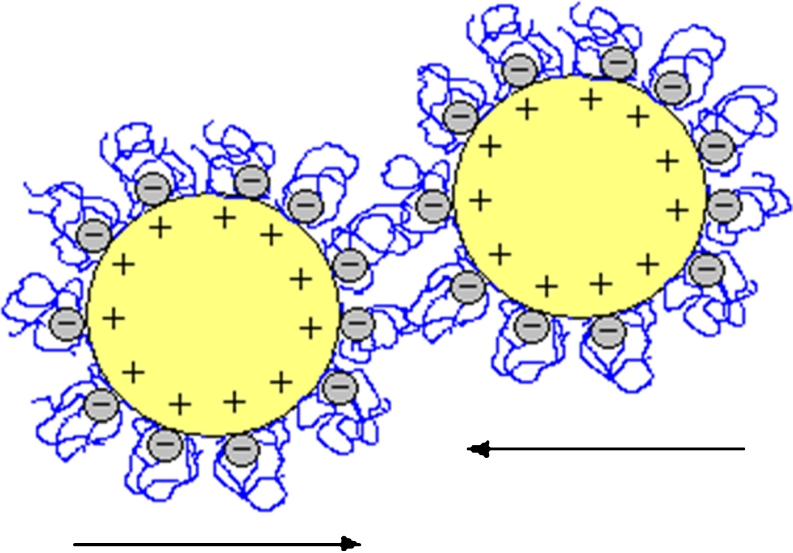
The mechanism of polymer bridges formation between Cr_2_O_3_ particles at pH = 3

The addition of the polymer to the chromium (III) oxide suspension at pH = 3 causes a significant decrease of system stability. Under these conditions, destabilization is primarily caused by the neutralization of the positive surface charge of metal oxide particles by the negatively charged PAA chains. As mentioned earlier, at pH <4.5, undissociated carboxylic groups dominate in the PAA macromolecules, but the number of dissociated groups is sufficient to effectively neutralize the solid particle charge. On the other hand, a small number of dissociate groups in the PAA chains causes more coiled conformation. During the PAA adsorption on the chromium (III) oxide surface, the –COO^−^ groups interact with the positive surface of solid particles. This leads to almost complete particle coverage by the coiled polymer chains (the highest adsorption of PAA). The adsorbent surface charge neutralization by the adsorbed PAA chains at pH = 3 is schematically shown in Fig. 9. The results of zeta potential measurements obtained under the same conditions (Fig. 7) are the confirmation of this destabilization mechanism. Adsorption of polymer on the Cr_2_O_3_ surface shifts the pH_iep_ value of these solid particles to pH = 4 (PAA 2,000) or pH = 3 (PAA 240,000). Close to pH_iep_, metal oxide particles are coated with the polymer and as a result they cease to repel, which allows their collision and flocs formation (solid charge neutralization by adsorbed polymer). In addition, at pH = 3 the polymer bridges can be formed. They also cause suspension destabilization. Polymer bridges are formed through the interaction of two different segments of the polymer chains adsorbed on the surface of two different colloidal particles. The mechanism of polymer bridges formation between the Cr_2_O_3_ particles is shown in Fig. 10.

**Fig. 11:**
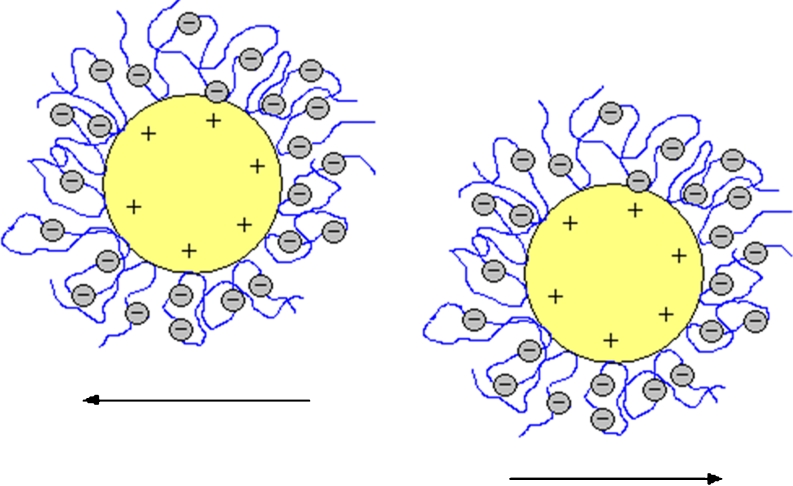
The electrosteric stabilization of Cr_2_O_3_ particles in the presence of PAA at pH = 6

At pH = 6, the addition of polyacrylic acid significantly improves the stability of the Cr_2_O_3_ suspension. Under these conditions, there is the electrosteric stabilization resulting from the sum of the electrostatic repulsion between negative charges of the adsorbed macromolecules and the steric repulsion of polymer layers. Thus, there is electrosteric repulsion between the colloidal particles coated with negatively charged polymer layers which make the system stable. Since at pH = 6 most of the carboxylic groups of polymer chains is dissociated (α_d_ = 0.96) and the repulsion between the polymer segments occurs, the PAA chains have more expanded structure. Moreover, under these conditions the Cr_2_O_3_ surface charge density decreases, and as a result, only a small number of polymer chains containing –COO^−^ groups interact with a solid surface (the average amount of polymer adsorption). The other ionized PAA carboxylic groups within the loop and tail structures cause the electrosteric stabilization of the Cr_2_O_3_ suspension, as schematically shown in Fig. 11.

**Fig. 12:**
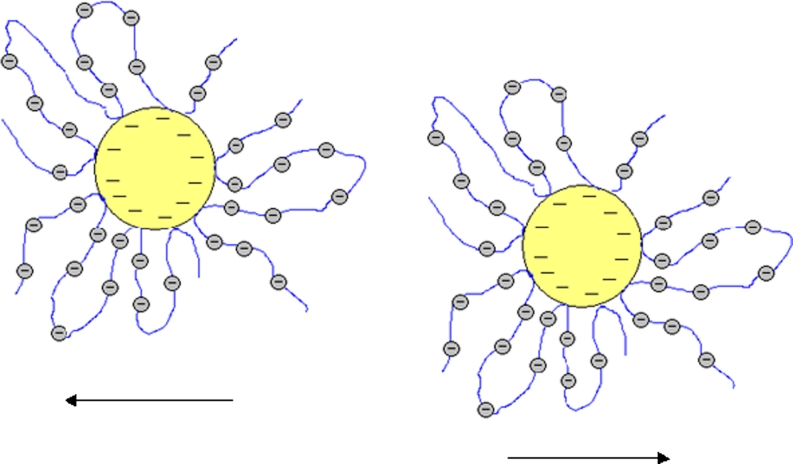
The electrosteric repulsion of Cr_2_O_3_ particles coated by PAA chains at pH = 9

At pH = 9 the polymer addition to the suspension results in an evident increase of the system stability. In this case, as at pH = 6, electrosteric stabilization occurs (large absolute values of zeta potential in the range from −60 to −70 mV independent of the PAA concentration) in the system. At pH = 9, the particle surface is negative and a large number of dissociated carboxylic groups results in even stronger repulsion of polymer segments and greater extension of the chains than at pH = 6. Electrostatic repulsion between the negatively charged solid surface and the negatively charged polymer segments makes the PAA adsorption specific. This process is only possible through the formation of hydrogen bonds. Besides, the strong adsorbent–adsorbate repulsion makes PAA adsorption the smallest under these pH conditions. The structure of polymer adsorption layer, which causes the effective electrosteric stabilization at pH = 9, is shown schematically in Fig. 12.

## Conclusions

The obtained results show that: (1) the maximum adsorption of PAA on the chromium (III) oxide surface occurs at pH = 3, (2) during PAA adsorption on Cr_2_O_3_ surface the hydrogen bridges are formed, (3) the presence of PAA in the system lowers the zeta potential of Cr_2_O_3_ particles and reduces their surface charge, (4) reduction of Cr_2_O_3_ zeta potential in the presence of PAA is due to the nature of the anionic PAA, shift of slipping plane and blocking the active sites on the Cr_2_O_3_ particles surface, (5) Cr_2_O_3_ suspension not containing PAA is the most stable at pH = 3 (electrostatic stabilization) and (6) Cr_2_O_3_ suspension containing PAA is the most unstable at pH = 3 (solid charge neutralization by the adsorbed polymer).

To sum up, polyacrylic acid at pH 3 proved to be the most effective flocculant of the chromium (III) oxide suspension. The use of this information in the development of effective methods for chromium (III) oxide removal from water would provide the greatest possible aggregates of the oxide particles as soon as possible. The electrosteric stabilization compared with the electrostatic stabilization is distinguished by several advantages. These include low sensitivity to the presence of electrolytes, comparable efficacy in aqueous and non-aqueous environment, comparable efficacy at low and high concentrations of colloidal particles and the reversibility of flocculation.
